# Lamprey-Inspired Amphibious Suction Disc with Hybrid Adhesion Mechanism

**DOI:** 10.34133/cbsystems.0527

**Published:** 2026-02-24

**Authors:** Lei Li, Wenzhuo Gao, Boyang Qin, Yiyuan Zhang, Changhong Linghu, Bo Wang, Yitian Ma, Shihan Kong, Junzhi Yu

**Affiliations:** ^1^ Institute of Ocean Research, Peking University, Beijing 100871, China.; ^2^ School of Advanced Manufacturing and Robotics, Peking University, Beijing 100871, China.; ^3^ College of Design and Engineering, National University of Singapore, Singapore 119077, Singapore.; ^4^ Department of Mechanical Engineering, City University of Hong Kong, Hong Kong SAR 999077, China.; ^5^ School of Mechanical and Electrical Engineering, Beijing Institute of Technology, Beijing 100081, China.

## Abstract

Bioinspired adhesives mimicking octopuses, tree frogs, and geckos enable robots to grip and manipulate diverse surfaces. However, most existing systems use a single adhesion mechanism, limiting adaptability and hindering strong, reversible attachment across diverse surface conditions and environmental media. Here, inspired by the oral sucker of the lamprey (*Lethenteron reissneri*), we present a hybrid suction disc that integrates a thermally switchable shape-memory polymer (SMP) panel for surface conformity and a soft silicone lip for vacuum suction. When heated, the SMP softens to conform to surface irregularities; subsequent cooling restiffens it, enabling mechanical interlocking with surface asperities under vacuum. This synergistic design achieves robust, reversible, and cross-medium adhesion on challenging surfaces, both in air and underwater. The disc generated peak pull-off forces of 562 N in air and 590 N underwater on smooth substrates, over 850 times its own weight, and maintained strong adhesion even on rough surfaces (>707 μm) where conventional suction fails. Incorporating the SMP improved adhesion by 377% in air and 270% underwater compared to vacuum alone. Shear friction tests showed similar enhancements, and attachments remained secure for 26.8 h under load. The hybrid disc also enabled robotic demonstrations of gripping and cross-medium manipulation when mounted on a mechanical arm, highlighting its potential for real-world robotic applications. This work paves the way for developing multimodal adhesion systems and amphibious robots capable of adaptive gripping and reliable operation across diverse environments.

## Introduction

Adhering reliably to surfaces in diverse environments, including dry, wet, and fully submerged conditions, remains a long-standing challenge for climbing robots [1,2], mobile manipulators [3,4], and robots operating across multiple media [5]. To address this, researchers have long turned to biological systems for inspiration. In nature, a wide array of terrestrial and aquatic animals, such as geckos [6], tree frogs [7], birds [8], octopuses [9], and remoras [10], have independently evolved specialized adhesion structures to survive in complex and dynamic environments. These biological systems exhibit reversible, adaptable, and energy-efficient adhesion strategies tailored to specific environmental and surface conditions.

Biological adhesion mechanisms are typically categorized into 4 types: (a) dry adhesion, based on van der Waals forces between micro/nanostructures and substrates, as seen in gecko toe pads [11]; (b) wet adhesion, which relies on capillary forces from mucus or fluid films, as used by tree frogs [12]; (c) mechanical interlocking, where claws or spines engage with surface irregularities, common in birds and insects [13]; and (d) suction-based adhesion, where soft tissues form sealed cavities to generate negative pressure, exemplified by octopuses and remoras [14]. These 4 mechanisms cover the principal physical strategies of biological adhesion. Each has inspired robotic implementations, yet each operates optimally only under specific constraints.

In robotics, for example, gecko-inspired dry adhesive pads have enabled machines such as the StickyBot to scale smooth vertical surfaces such as glass or ceramic [15]. Similarly, tree-frog-inspired wet adhesive pads can grip soft, mucus-covered tissues, which is especially useful in biomedical or wet environments [16]. Both strategies, however, degrade underwater, as thin water layers disrupt van der Waals forces in dry adhesives and wash away the capillary bridges that wet adhesion relies on. By contrast, mechanical interlocking (microspine grippers) and suction-based systems (octopus-inspired suckers) can function in both air and water environments [17–19], but each has limitations: Interlocking requires rough surfaces, while suction is ineffective on porous or uneven substrates. These trade-offs have spurred interest in hybrid adhesion strategies that combine multiple mechanisms for enhanced adaptability across diverse surfaces and environmental conditions.

**Fig. 1.**
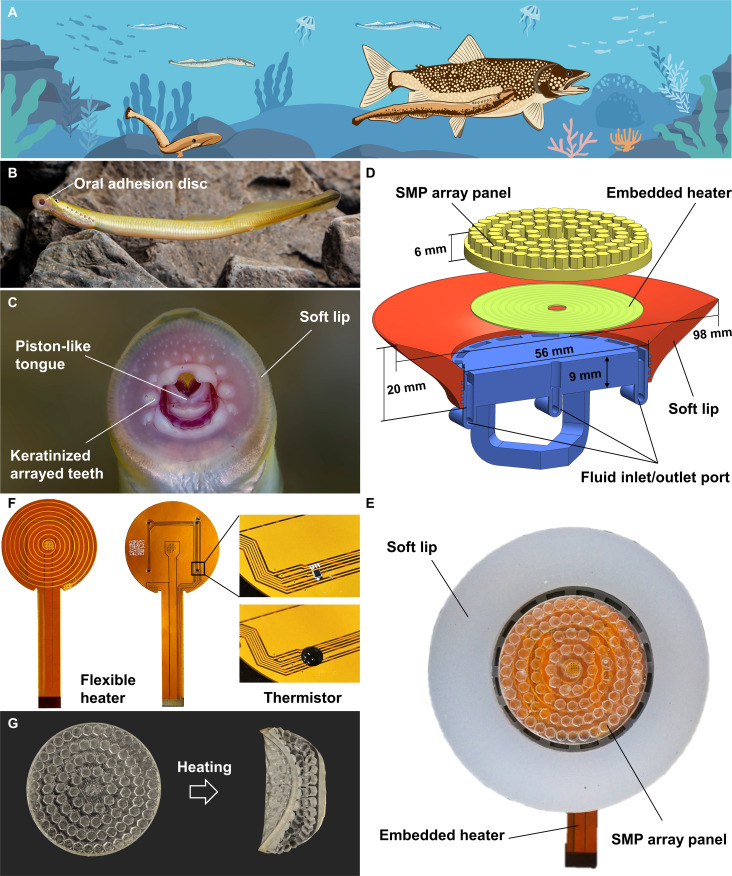
Bioinspired design and prototype of an SMP-enhanced amphibious suction disc. (A) Schematic of a lamprey attaching to rocky substrates and the surface of a host fish using its oral suction disc. (B) Live lamprey exhibiting its native adhesive disc (*Lethenteron reissneri*). Photo credit: H. Chen. (C) Ventral close-up of the lamprey’s disc, showing soft periphery and keratinized teeth. (D) CAD schematic of the robotic suction disc: SMP array panel (yellow), embedded resistive heater (green coil), soft silicone lip (red), and rigid base with fluid inlet/outlet ports (blue). (E) Top view of the assembled robotic disc. (F) Flexible printed heater incorporating 4 embedded temperature sensors. (G) SMP array panel: left, glassy state before heating (rigid); right, rubbery state after heating (soft and deformable).

Nature provides a compelling example of such integrated adhesion in the lamprey (Petromyzontidae), a jawless aquatic vertebrate that evolved over 500 million years ago [20]. The lamprey’s circular oral disc features a soft, flexible lip that conforms to lowly rough surfaces, a muscular pump that generates strong negative pressure, and multiple inward-facing keratinized teeth that mechanically interlock with its host (Fig. 1A to C) [21,22]. This combination enables secure, reversible, and energy-efficient attachment, even in turbulent flow. Despite these advantages, the lamprey’s synergistic suction-interlocking mechanism remains largely underutilized in robotic adhesion systems.

**Fig. 2.**
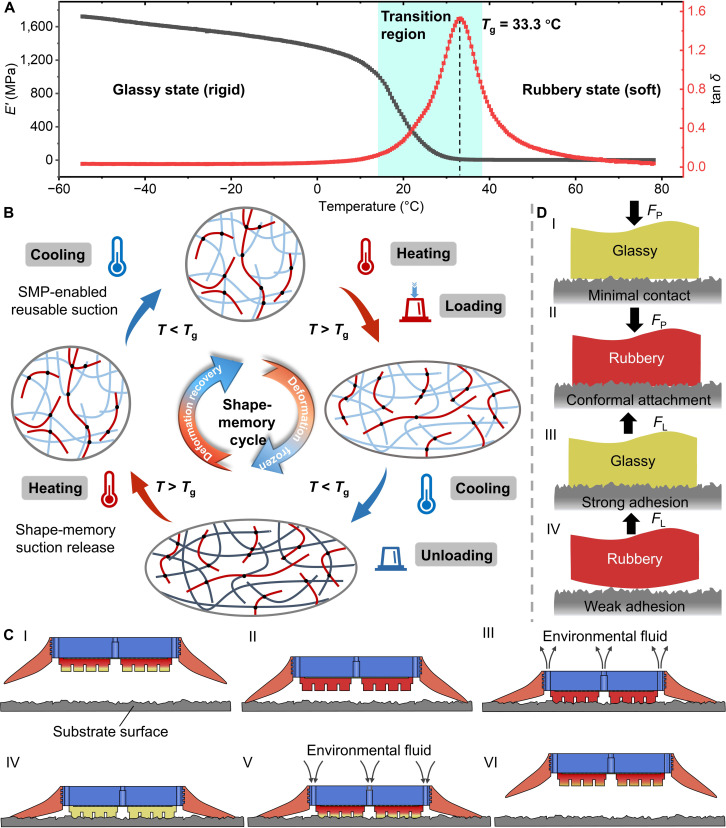
Working principle of the bioinspired suction disc for reversible attachment and detachment. (A) Dynamic mechanical analysis of the SMP panel showing storage modulus *E′* (black) and loss tangent tan *δ* (red) versus temperature. The glass transition temperature (*T*_g_ = 33 °C) marks the transition from a rigid glassy state to a soft rubbery state. (B) Molecular schematic of the SMP shape-memory effect. Above *T*_g_, the reversible-phase chains become mobile and rearrange under loading to encode a temporary shape; cooling below *T*_g_ freezes chain mobility and locks this shape, while reheating above *T*_g_ unlocks the network and drives recovery to the permanent shape. Black dots denote cross-linking netpoints; red lines represent stationary-phase chains; light-blue lines represent reversible-phase chains; dark-blue lines indicate chains frozen below *T*_g_. (C) Attachment and release sequence. (I) Heat the SMP panel and approach the surface. (II) Soft lip and substrate form a seal. (III) Fluid expulsion generates negative pressure adhesion, pressing the SMP panel onto the surface. (IV) SMP cools and interlocks with the surface. (V) Reheat the SMP and allow fluid ingress to break the seal. (VI) Disc fully detaches. (D) Principle of SMP-panel attachment and detachment. (I) Minimal contact in the glassy phase (preload force *F*_P_). (II) Conformal attachment in the rubbery state under negative pressure actuation. (III) Strong adhesion after cooling back into the glassy phase (lifting force *F*_L_). (IV) Adhesion release by reheating into the rubbery state.

**Fig. 3.**
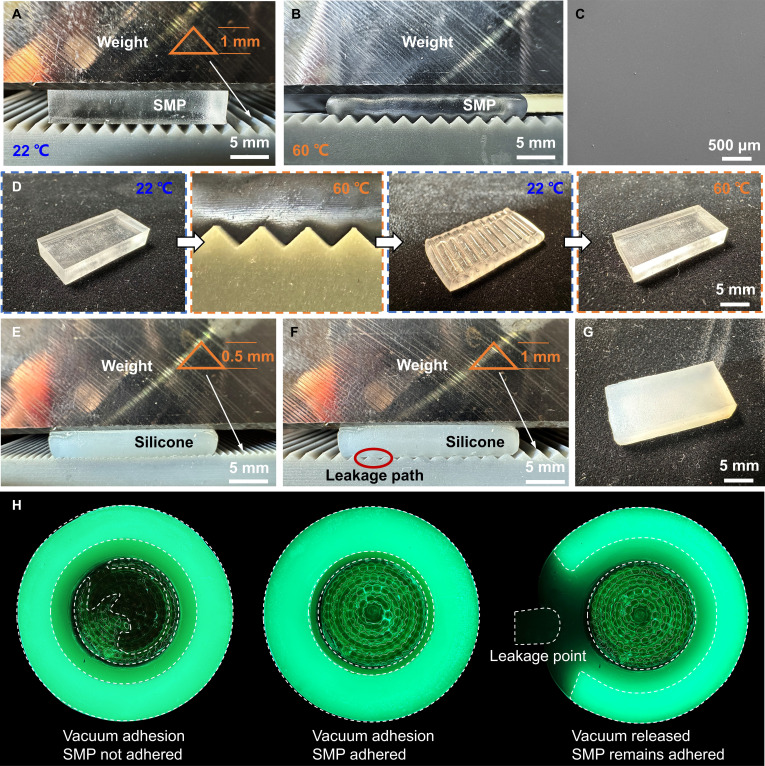
Hybrid suction enabled by thermally controlled interfacial locking and sealing. (A and B) Thermally switchable conformal contact of the SMP panel on a sawtooth substrate under identical preload. At 22 °C (glassy), the SMP remains rigid and makes limited contact, whereas, at 60 °C (rubbery), it softens and is pressed into surface asperities, imprinting the surface topography. (C) SEM image of the SMP panel surface in its initial state, showing a smooth morphology. (D) Shape-memory locking and recovery mechanism. Heating above *T*_g_ softens the SMP to enable surface imprinting, cooling below *T*_g_ rigidifies and locks the imprinted geometry to establish mechanical interlocking, and reheating unlocks the interface and drives recovery to the initial shape. (E to G) Silicone-only controls isolate the sealing contribution. Silicone forms a complete seal on 0.5-mm features but fails on 1-mm asperities, leaving an interfacial gap and leakage path on overly rough surfaces. The seal relies purely on elastic deformation and does not provide imprint locking after unloading. (H) FTIR contact maps resolve the hybrid mechanism (left to right): vacuum adhesion with SMP not engaged, combined vacuum and SMP adhesion, and adhesion maintained by SMP after vacuum release (Movie S1). Black indicates no contact, and green indicates contact.

One promising approach to bridging this bioinspiration gap is to integrate stimuli-responsive materials into adhesion devices. In particular, shape-memory polymers (SMPs) offer programmable deformation, shape retention (locking), and stiffness modulation in response to thermal stimuli [23,24]. When incorporated into an adhesive structure, an SMP can be heated to a soft, rubbery state to achieve close conformal contact with a surface and then cooled to return to a rigid, glassy state that locks the deformed shape in place (Fig. 2). This shape-locking effect effectively preserves intimate contact and enhances attachment stability. Moreover, SMP-based adhesives can be switched between high-adhesion and low-adhesion states on demand by toggling the material’s temperature, enabling controlled attachment and easy release (Fig. 3).

In this work, we present a lamprey-inspired suction disc that combines a thermally responsive SMP skeleton with a compliant outer lip to achieve robust adhesion in both air and water. The design integrates 2 complementary mechanisms: (a) an internal SMP-based framework that can lock its shape and stiffen when cooled; and (b) an external suction chamber that forms a seal and generates negative pressure for attachment. By combining active shape control with suction, this design emulates the lamprey’s dual adhesion strategy in an engineered system. We systematically evaluate how this hybrid approach influences adhesion strength, frictional force, and attachment duration, and we demonstrate its adaptability to surfaces with varying roughness. Our findings highlight the potential of multimodal, bioinspired adhesion systems for next-generation climbing robots, amphibious vehicles, and underwater manipulators operating across air–water interfaces.

## Materials and Methods

### Fabrication of the bioinspired lamprey disc

The suction disc was fabricated through sequential multimaterial molding and assembly. First, a rigid base was 3-dimensionally (3D) printed in a photopolymer resin. The soft silicone lip was formed by mixing Ecoflex 30 precursors (1:1 by weight), degassing, and casting the mixture into a 3D-printed mold seated on the base, followed by curing at room temperature for 4 h (Fig. S1A).

The integrated heating and temperature-sensing circuitry was implemented using a custom flexible printed circuit (FPC). The heater used a spiral trace (0.5-mm width and 2.4-mm pitch) designed in electronic design automation software to promote uniform heating. The FPC was fabricated (JLCPCB, China) with a copper foil thickness of 1/3 oz, achieving a nominal resistance of 2 Ω. To enable thermal feedback, an analog temperature sensor (TMP235, Texas Instruments) was integrated onto the FPC via surface-mount technology using high-temperature solder paste (solidus temperature of 238 °C). A layer of silicone sealant (Kafuter 704, China) was subsequently encapsulated over the sensor to provide robust mechanical fixation and waterproofing. This heater sensor FPC was subsequently embedded into the SMP panel during comolding.

To form the SMP microstructure, a rigid master mold defining the pillar array geometry was 3D-printed, and a compliant silicone mold (Ecoflex 30) was cast from the master to facilitate demolding. The FPC and SMP precursors were then comolded in the silicone mold. The SMP precursor consisted of E44 epoxy (Feicheng Deyuan Chemical Co., China) and poly(propylene glycol) bis(2-aminopropyl ether) curing agent (Shanghai Aladdin Bio-Chem Technology Co. Ltd., China) mixed at a 1:1.55 weight ratio and degassed for 30 min. The assembly was cured stepwise at 50 °C for 2 h, 100 °C for 1 h, and 130 °C for 1 h to complete polymerization and establish the shape-memory network (Fig. S1B).

After curing, the SMP panel was demolded and bonded to the base using Loctite HP-20 epoxy loaded with aerogel powder, followed by a 12-h room-temperature cure to accommodate thermal mismatch between the photopolymer base and the SMP. The resulting disc integrates a rigid support, a soft sealing lip, an actively deformable SMP panel, and embedded heating and sensing functions (Fig. S1C).

### Adhesion test setup for bioinspired lamprey disc

We measured adhesive and frictional stresses using a Mark-10 F105 universal testing machine equipped with a 1,000-N load cell on smooth acrylic plates and rough substrates (3M sandpaper). All tests were conducted at room temperature. For underwater measurements, we placed a custom acrylic tank (180 mm × 150 mm × 150 mm) on the testing machine base and filled it with water, whereas the same setup was used dry for tests in air. In each trial, the SMP panel was preheated for 1 min to reach 60 °C and fully enter the rubbery state. We then applied a prescribed internal pressure (vacuum level) using a feedback-controlled pump, held the setpoint for 1 min, and subsequently turned off both the pump and the heater. The disc was allowed to cool for 30 min in air or 15 min underwater before pull-off or shear testing. The crosshead speed was set to 100 mm/min. To quantify maximum adhesion duration on rough substrates (*R*_a_ = 25 and 52 μm), we applied constant normal loads of 5, 10, 15, and 20 N using a Zwick Z0.5 universal testing machine (Zwick/Roell, Ulm, Germany), maintained each load until spontaneous detachment, and recorded the time to failure. Using the same instrument, we also performed creep tests on SMP specimens (20 mm × 10 mm × 4 mm) under a constant load of 5 N and recorded the strain evolution over 24 h.

### Statistical analysis

**Fig. 4.**
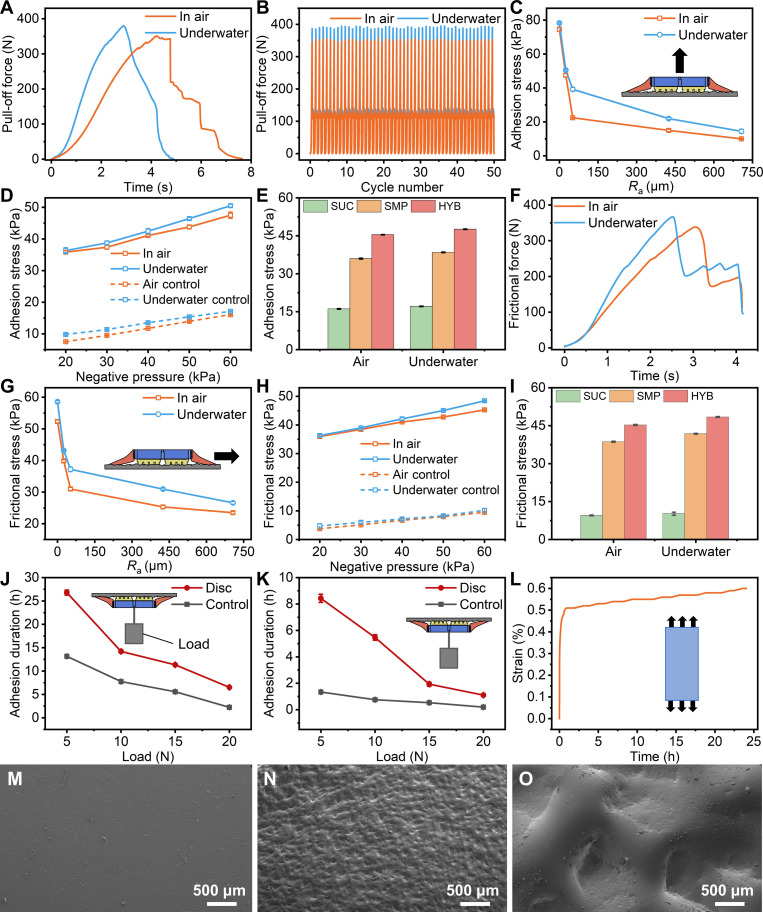
Adhesion performance of the lamprey-inspired disc in air and underwater. (A) Pull-off force versus time on a rough surface (*R*_a_ = 25 μm) in air and underwater (SMP panel heated to 60 °C for 1 min prior to testing). (B) Repeatability over 50 attachment–detachment cycles on the same rough substrate (*R*_a_ = 25 μm) in both environments. (C) Maximum adhesion stress of the disc on surfaces with varying roughness (*R*_a_ = 0 to 707 μm) in air and underwater (*N* = 5). (D) Adhesion stress as a function of applied negative pressure in both environments, compared with control discs in which the SMP panel was replaced by a rigid resin plate (*R*_a_ = 25 μm, *N* = 5). (E) Comparison of adhesion stress among 3 modes: negative pressure (SUC), SMP deformation (SMP), and the full disc (HYB), measured in air and underwater (*R*_a_ = 25 μm, *N* = 5). (F) Frictional force versus time on a rough surface (*R*_a_ = 25 μm) in air and underwater. (G) Maximum frictional stress of the disc on surfaces with different roughness levels (*R*_a_ = 0 to 707 μm) in air and underwater (*N* = 5). (H) Frictional stress as a function of negative pressure, compared with control discs without SMP panel (*R*_a_ = 25 μm, *N* = 5). (I) Comparison of frictional stress among 3 modes (SUC, SMP, and HYB) measured in air and underwater (*R*_a_ = 25 μm, *N* = 5). (J) Adhesion duration of the biomimetic disc and control group under different loads in air (*R*_a_ = 25 μm, *N* = 3). (K) Adhesion duration of the biomimetic disc and control group underwater (*R*_a_ = 50 μm, *N* = 3). (L) Creep response of the SMP at room temperature under a constant load of 5 N (specimen, 20 mm × 10 mm × 4 mm), showing the strain evolution over 24 h. (M to O) SEM images of interfacial contact features. (M) Silicone surface morphology. (N) SMP surface after thermal imprinting and locking against a moderately rough substrate (*R*_a_ = 50 μm). (O) SMP surface after thermal imprinting and locking against a highly rough substrate (*R*_a_ = 707 μm). All error bars represent ±1 SD.

Error bars represent ±1 SD. One-way (Fig. 4E and I) and 2-way (Fig. 4C, D, G, H, J, and K) analyses of variance (ANOVA) were conducted to evaluate the statistical significance of the experimental results. The significance threshold was set at *P* < 0.05. All statistical analyses were performed using OriginPro (version 2022, OriginLab, USA).

## Results

### Design and fabrication of bioinspired lamprey disc

On the basis of the morphological features of the natural lamprey’s suction disc, we designed and fabricated a bioinspired prototype (Fig. 1D and E). The prototype measures 98 mm in diameter and 40 mm in height, weighing 70 g. It comprises a 3D-printed rigid base, a soft silicone lip, a flexible printed heater circuit, and an SMP array panel. The rigid base features dovetail grooves that interlock with the soft lip, which substantially enhances the bonding between these materials.

We patterned a spiral heating circuit (0.5-mm trace width and 2-mm pitch) onto a flexible elastomer substrate and integrated 4 temperature sensors in a quadrilateral array to enable asymmetric heating (Fig. 1F). The flexible circuit’s intrinsic compliance ensures stable conductivity even through repeated deformations of the SMP elastomer. Meanwhile, the 4-sensor arrangement effectively monitors heating uniformity across the surface. Each ultraminiature sensor (0.88 mm × 0.88 mm) was encapsulated in silicone sealant to ensure secure, long-term operation without exposure to the environment.

In its glassy (rigid) state, the SMP panel is dimensionally stable and provides high stiffness and structural support under load. When heated above its transition temperature into the rubbery state, the SMP becomes soft and highly elastic, allowing large but fully recoverable deformations. We also designed a hexagonal array of small pillars (2-mm side length and 3-mm height) on the SMP panel, leaving interstitial gaps between pillars to improve the material’s ability to conform to surface irregularities in the rubbery state (Fig. 1G).

### Working principle of the bioinspired lamprey disc

Dynamic mechanical analysis of the SMP panel revealed a pronounced glass transition at *T*_g_ = 33 °C. At this transition temperature, the storage modulus (*E′*) drops sharply from approximately 1,600 MPa in the glassy state to less than 5 MPa in the rubbery state. The loss tangent (tan*δ*) simultaneously shows a distinct peak, indicating maximal polymer chain mobility during the phase transition (Fig. 2A). At the molecular level, the SMP is a covalently cross-linked network comprising a stationary phase that defines the permanent shape and a reversible phase that acts as a thermal switch (Fig. 2B). Above *T*_g_, reversible-phase chains become mobile and rearrange under preload, enabling conformal deformation and surface imprinting. Cooling below *T*_g_ freezes chain mobility and fixes the imprinted geometry as a temporary shape. Reheating above *T*_g_ restores chain mobility, releases the locked deformation, and drives recovery to the permanent shape for repeatable resetting. In the schematic, black dots denote cross-linking netpoints, red lines represent stationary-phase chains, light-blue lines represent reversible-phase chains, and dark-blue lines indicate chains frozen below *T*_g_. Collectively, this thermally switchable stiffness enables the disc to alternate between a compliant state for conformal imprinting and a stiff state for geometric locking.

The working mechanism of the bioinspired suction disc relies on the coordinated interaction between the SMP panel and the pressure regulation system (Fig. 2C-I to VI). First, resistive heating softens the SMP into its rubbery state (Fig. 2C-I). The disc is then brought into contact with the target substrate. The soft silicone lip conforms to the surface, creating a fluid-tight seal (Fig. 2C-II). Next, we evacuate fluid from the internal chamber to expel air (or water), generating a negative pressure. This vacuum presses the still-rubbery SMP panel firmly into the substrate’s microtopography, achieving intimate, conformal contact (Fig. 2C-III and D-II). Upon cooling, the SMP reenters its glassy, rigid phase and mechanically interlocks with surface asperities, dramatically increasing adhesion strength (Fig. 2C-IV and D-III). To release the disc, we reheat the SMP back to the rubbery state, while we admit the ambient fluid into the chamber to break the seal and collapse the vacuum (Fig. 2C-V and D-IV), enabling rapid detachment and reset (Fig. 2C-VI). This cycle of conformal attachment under vacuum in the rubbery state, followed by strong interlocked adhesion after resolidification, and finally on-demand release by reheating, yields a robust and switchable adhesion mechanism. The system functions effectively in both dry and submerged environments. Notably, if the SMP was applied in its glassy state without this process, direct contact yields only minimal contact area and negligible adhesion on most surfaces (Fig. 2D-I).

To experimentally substantiate the proposed hybrid suction mechanism, we decoupled and visualized the thermally controlled interfacial locking of the SMP and the sealing behavior of the silicone lip (Fig. 3). Under identical preload on a sawtooth substrate, the SMP exhibited strongly temperature-dependent conformity. At 22 °C, the glassy SMP remained rigid and contacted only the asperity tips, indicating limited conformal engagement (Fig. 3A). In contrast, when heated to 60 °C, the SMP softened into the rubbery state and was driven into the surface grooves, producing clear topographic imprinting (Fig. 3B). Scanning electron microscopy (SEM) imaging confirmed that the SMP surface was initially smooth, excluding preexisting texture as the origin of the observed imprint patterns (Fig. 3C). A complete shape-memory imprint–lock–recover cycle further revealed the locking mechanism. Heating above *T*_g_ enabled imprint formation, subsequent cooling below *T*_g_ rigidified the deformed geometry to lock the imprint and establish mechanical interlocking, and reheating erased the lock and restored the original surface (Fig. 3D).

We next isolated the sealing contribution using silicone-only controls. The compliant silicone could elastically deform to form a complete seal against moderate features (0.5 mm), whereas larger asperities (1 mm) prevented full closure and left an interfacial gap that served as a leakage path on overly rough surfaces (Fig. 3E and F). Importantly, this sealing effect relied solely on elastic deformation and did not retain an imprinted geometry after unloading, underscoring the absence of interfacial locking in silicone alone (Fig. 3G). Finally, frustrated total internal reflection (FTIR) contact imaging provided direct evidence for the hybrid mechanism at the disc scale. When the SMP remained in the glassy (unheated) state, the disc showed poor conformity to the surface, resulting in incomplete contact. Once the SMP was heated into the rubbery state, it softened and conformed closely to the substrate, enabling full-area contact under the applied negative pressure. After the SMP was cooled back into its glassy state, that conformal shape was “locked” in place, providing strong and durable adhesion. Importantly, even after the external vacuum was released, the interlocked SMP layer maintained intimate contact with the substrate (Movie S1). This prolonged the adhesion time well beyond what vacuum suction alone could achieve. This synergistic mechanism effectively decouples the adhesion strength from continuous vacuum maintenance and demonstrates the potential of SMP-assisted discs for long-duration attachment.

### Electrical and heating control of the bioinspired lamprey disc

The integrated heating vacuum control hardware and electrical architecture are summarized in Fig. S2, the closed-loop regulation scheme is shown in Fig. S3A, and the corresponding control logic is detailed in Text S1. We then characterized the heating performance in air and underwater. Infrared thermography confirmed spatially uniform heating of the SMP panel and stable temperature regulation at a 60 °C setpoint (Fig. S3B). We recorded temperature versus time at setpoint temperatures of 40, 60, and 80 °C in each medium. In air, the disc reached 60 °C in 2.76 ± 0.04 s. Underwater, the suction disc design provides some insulation for the heater, allowing the flexible heating element to reach 60 °C in 5.33 ± 0.06 s and thus rapidly activate the SMP (Fig. S3C). The maximum temperature overshoot at these setpoints did not exceed 1.5% in either medium. As expected, the heating time increased with higher temperature setpoints, and heating was consistently slower underwater due to water’s high heat capacity and convective cooling (Fig. S3D). For example, reaching 80 °C underwater required 11.71 ± 0.04 s, which is roughly twice as long as in air (approximately 109.7% increase in heating time).

### Adhesion performance of the bioinspired lamprey disc

We quantitatively characterized the adhesion and frictional performance of the lamprey-inspired suction disc in both air and underwater environments (Fig. 4). The system integrates a control board, fluidic pump, valve, pressure sensor, communication module, and power supply to autonomously regulate suction (Fig. S4). The pressure sensor continuously monitors the internal cavity pressure and adjusts suction strength in real time through pump feedback control. In each test, the SMP panel was heated to 60 °C to fully soften for conformal contact and then allowed to cool after attachment to return to the glassy state and lock the interfacial geometry, thereby stabilizing adhesion.

We first measured time-resolved pull-off force curves on a moderately rough surface (surface roughness *R*_a_ = 25 μm) both in air and underwater (Fig. 4A). During these tests, the chamber gauge pressure was regulated to −60 kPa (relative to ambient) to provide consistent suction loading. In air, the disc’s maximum pull-off force reached about 350 N. Underwater, the disc achieved a higher and broader force peak, reaching about 379 N. The overall shape of the force–time curve was similar in both cases, as one adhesion mechanism gave way first and was followed shortly by the other, but the peak forces differed, with underwater performance being superior. We then assessed operational stability by running 50 consecutive attachment–detachment cycles on the same substrate (*R*_a_ = 25 μm) in both environments (Fig. 4B). Across repeated cycles, the pull-off response remained highly consistent, indicating robust repeatability of the disc’s attachment state and release process. Importantly, the peak pull-off force showed only small cycle-to-cycle variation and no obvious degradation trend over the 50 cycles in either medium, demonstrating stable performance under repeated use on rough surfaces.

The maximum adhesion stress, defined as the maximum pull-off force divided by the disc area, decreased monotonically with increasing surface roughness in both air and water (Fig. 4C, *P* < 0.05). On smooth substrates, the disc achieved adhesion stresses of 74.6 ± 1.0 kPa (562.6 ± 8.0 N) in air and 78.3 ± 0.5 kPa (590.7 ± 3.5 N) underwater. As surface roughness increased, the adhesion stress gradually declined, reaching 9.9 ± 0.3 kPa in air and 14.5 ± 1.2 kPa underwater at *R*_a_ = 707 μm. In air, adhesion dropped sharply once *R*_a_ exceeded about 52 μm because the silicone lip could no longer maintain a vacuum seal on very rough surfaces, leading to rapid loss of suction during pull-off process. In contrast, at that same roughness underwater, the surrounding water (incompressible) helped stabilize the lip seal, so negative pressure remained effective and adhesion stayed substantially higher. Beyond roughly *R*_a_ = 25 μm in air and 52 μm underwater, the majority of the remaining adhesion was provided by the SMP interlocking with the surface rather than by vacuum suction. Notably, the SMP’s functional roughness limit extends well beyond *R*_a_ = 707 μm, as later tests on highly irregular objects demonstrated. Overall, adhesion in water consistently exceeded that in air across all roughness levels, owing to the incompressibility of the surrounding fluid, which stabilizes the seal of the silicone lip, and to the enhanced interfacial wettability and conformal contact of the SMP, which strengthen solid–solid adhesion even without negative pressure.

The lamprey-inspired suction disc fundamentally differs from a conventional suction cup by incorporating an internal SMP adhesion layer that mimics the tooth-like microstructures in real lamprey mouths. To isolate the effect of this SMP layer, we compared adhesion performance with and without the SMP panel under identical vacuum conditions. We varied the applied negative pressure from −20 to −60 kPa and observed that, in both air and water, the adhesion stress increased approximately linearly with increasing vacuum (Fig. 4D, *P* < 0.05). However, when we replaced the SMP panel with a rigid resin plate (creating a control device that relied only on suction), the achieved adhesion was much lower under the same conditions. This confirms that the SMP’s deformation is crucial for enhancing attachment. In fact, the presence of the SMP layer boosted the maximum adhesion by up to 377% in air and 270% underwater compared to the rigid control, underscoring the SMP’s strong contribution to interfacial conformity and load sharing even in the presence of vacuum suction.

Because suction-only adhesion is ultimately limited by sealing, we further examined how lip stiffness affects sealing-based suction in the absence of SMP locking (Fig. S5). Suction cups were cast from a softer silicone (Ecoflex 00-30; manufacturer-reported 100% modulus, 69 kPa) and a stiffer silicone (Mold Star 30; 100% modulus, 662 kPa), and tested across substrates of different roughness in air and underwater. In both environments, the stiffer silicone achieved higher adhesion on smoother surfaces but dropped more sharply as roughness increased. The softer silicone maintained adhesion on rougher surfaces, albeit with lower peak values, highlighting a stiffness–adaptability trade-off for sealing lip design.

To further clarify the dominant adhesion mechanism, we tested 3 configurations at a surface roughness of *R*_a_ = 25 μm under a fixed vacuum of −60 kPa (in both air and water): a pure negative pressure suction cup (SUC; vacuum only and no SMP layer), a pure SMP attachment (SMP only and no active vacuum), and the hybrid suction disc (HYB; our device integrating both SMP and vacuum). In all cases, the hybrid disc achieved the highest adhesion strength, followed by the SMP-only configuration, while the vacuum-only cup showed the weakest attachment (Fig. 4E, *P* < 0.05). These results confirm that the SMP layer plays the primary role in generating strong adhesion. In addition, observations during detachment revealed that the SMP’s grip fails first (once it fractures or peels away from the surface), and the loss of the vacuum seal follows afterward. This sequential failure is consistent with the staged force–time behavior shown earlier (Fig. 4A). Moreover, combining negative pressure with the SMP layer provided a further benefit: The vacuum helped distribute tensile stresses more uniformly across the interface during pull-off. This prevented premature edge peeling or localized tearing of the SMP layer and thereby increased the detachment force. We measured that using vacuum together with SMP increased the peak adhesion force by roughly 26% in air and 23% underwater compared to SMP alone. Consequently, the hybrid suction disc, which synergistically integrates both mechanisms, exhibited the highest overall adhesion strength among all configurations tested.

In addition to strong normal (pull-off) adhesion, the lamprey-inspired disc demonstrated remarkable resistance to shear loading. In friction tests, the time-dependent frictional force curves (Fig. 4F) exhibited a characteristic stick–slip behavior. Specifically, as a shear force was applied, the frictional force would rise to a peak, then one adhesion mode would give way (causing a drop in force), and the other mode would continue to resist until complete detachment occurred. We measured the maximum frictional stress (peak frictional force divided by disc area) on surfaces of varying roughness in both air and water. On smooth substrates, the peak frictional stress reached 52.3 ± 0.5 kPa (corresponding to 394.7 ± 3.5 N of lateral force) in air and 58.6 ± 0.4 kPa (441.7 ± 2.4 N) underwater. With increasing surface roughness, the frictional stress gradually decreased, but even at *R*_a_ = 707 μm, it remained as high as 23.5 ± 0.4 kPa in air and 26.6 ± 0.5 kPa underwater (Fig. 4G, *P* < 0.05). Similar to the normal adhesion results, the frictional (shear) resistance increased roughly linearly with applied negative pressure. Replacing the SMP layer with a rigid plate caused the frictional stress to drop by up to 89% in air and 87% underwater (Fig. 4H, *P* < 0.05). This comparison confirms that the SMP’s ability to deform and interlock is the dominant factor in shear resistance. Among the configurations, the hybrid disc achieved the greatest frictional strength overall. For example, at *R*_a_ = 25 μm, the hybrid disc’s peak frictional stress was, on average, about 16% higher than that of the SMP-only configuration and about 377% higher than that of the vacuum-only configuration (Fig. 4I, *P* < 0.05).

Long-term adhesion tests under sustained loads further verified the stability of the suction disc’s attachment. In both air and water, the attachment duration decreased with increasing external load, but, in all cases, the SMP-assisted disc maintained adhesion for far longer than the control device (without SMP). In air, on a rough surface (*R*_a_ = 25 μm), our SMP-enhanced disc maintained a stable attachment for 26.8 ± 0.5 h under a 5-N load and for 6.5 ± 0.1 h under a 20-N load. These times represent up to a 195% increase compared with the control device at the same loads (Fig. 4J, *P* < 0.05). Underwater (*R*_a_ = 52 μm), the adhesion duration exceeded 8.4 ± 0.3 h at 5 N and remained 1.1 ± 0.1 h even under a 20-N load (Fig. 4K, *P* < 0.05). This corresponds to a maximum improvement of about 540% over the control in the 5-N load case. This enhanced durability is attributed to the SMP layer’s ability to preserve its interlocked microtopography upon reglassing, effectively preventing rapid seal relaxation after the negative pressure is released and maintaining long-term adhesion stability. After long-term adhesion, the SMP pillar array showed noticeable geometric distortion, with shortened pillars and enlarged cross-sections (Fig. S6, left). Importantly, a brief reheating step restored the pillar geometry toward its initial state (Fig. S6, right), indicating that the deformation is recoverable and that the SMP array can be reused over repeated long-duration locking cycles.

To evaluate the SMP’s mechanical stability at room temperature (20 to 25 °C), we performed a 24-h creep test under a constant tensile force of 5 N using a specimen of 20 mm × 10 mm × 4 mm (Fig. 4L). The strain increased by 0.6% over 24 h without abrupt softening, indicating stable SMP behavior at ambient temperatures below *T*_g_. To visualize the surface-level evidence of imprinting and locking, we further examined the materials by SEM (Fig. 4M to O). The silicone surface exhibited a comparatively smooth morphology without distinct imprinted features (Fig. 4M). By contrast, after thermal imprinting and locking against a rough substrate, the SMP surface retained clear roughness-dependent imprinted microtopography. Imprints formed on the moderately rough substrate (*R*_a_ = 50 μm) were evident on the SMP surface (Fig. 4N), whereas locking against the highly rough substrate (*R*_a_ = 707 μm) produced more pronounced and heterogeneous surface deformation features (Fig. 4O), consistent with enhanced geometric engagement on rougher surfaces.

### Adhesion demonstration of the bioinspired lamprey disc

**Fig. 5.**
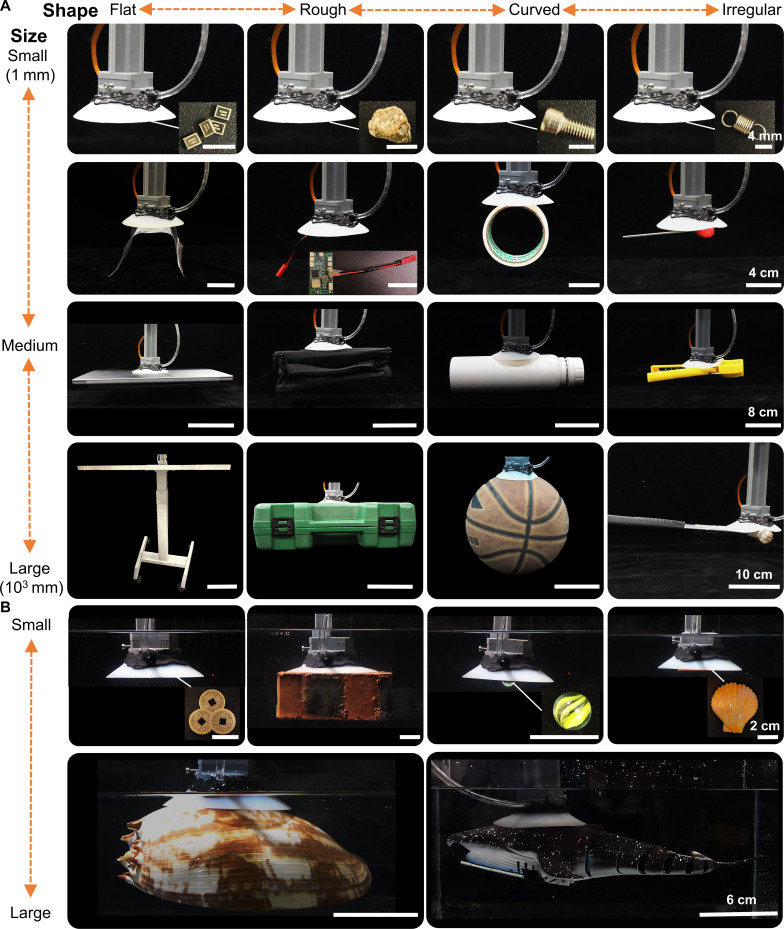
Versatile adhesion and manipulation performance of the biomimetic suction disc on diverse objects of varying size, shape, and surface texture in air and underwater. (A) In air, flat objects: electronic chips (0.01 g), a soft sealing bag (1.92 g), a laptop (1.10 kg), and a tabletop (11.4 kg); rough objects: a small stone (0.34 g), a circuit board (7.07 g), a camera bag (105.37 g), and a toolbox (1.52 kg); curved objects: a screw (0.66 g), a tape roll (80.20 g), a glass cup (481.40 g), and a basketball (606 g); irregular objects: a metal spring (0.34 g), a wrench (28.15 g), a pair of pliers (129.92 g), and a hammer (708 g) (Movie S2). (B) Underwater, flat objects: ancient coins (1.45 g); rough objects: a red brick (1.52 kg); curved objects: a glass sphere (3.54 g); irregular objects: a scallop shell (4.23 g), a conch shell (324.81 g), and a fish model (207.24 g) (Movie S3). Scale bars indicate the size of the adhered objects and are identical for all panels within each row (shown on the right).

Finally, to evaluate the adaptability of the bioinspired lamprey suction disc across diverse surfaces and environments, we performed a series of adhesion demonstrations on objects with different shapes and sizes in both air and underwater (Fig. 5 and Movies S2 and S3). We tested flat, rough, curved, and irregular surfaces, with object sizes ranging from the millimeter scale up to the meter scale. The disc exhibited robust attachment on all tested objects, maintaining stable adhesion even on highly uneven geometries.

In air, the disc securely gripped objects spanning over 6 orders of magnitude in mass (Fig. 5A). These ranged from tiny items such as electronic chips (0.01 g) and small stones (0.34 g), everyday objects such as a tape roll and a wrench, and even large items such as a laptop computer (1.1 kg) and a wooden tabletop (11.4 kg). The disc maintained reliable adhesion across a variety of surface morphologies: smooth planar surfaces (chip and laptop), rough or textured surfaces (circuit board and toolbox exterior), curved objects (water cup and basketball), and irregularly shaped tools (wrenches and hammers).

Underwater, the suction disc performed with similarly strong and versatile adhesion (Fig. 5B). It attached effectively to both natural and artificial substrates over a comparable mass range, from small objects such as an ancient coin (1.45 g) to larger, irregularly shaped items including scallop shells and model fish specimens weighing over 200 g. The disc gripped smooth metallic surfaces underwater, as well as rough, porous, or curved targets such as a red brick, scallop shells, and a large conch shell. Notably, the disc’s underwater performance was consistent, despite substantial variation in object curvature and surface texture. Overall, these demonstrations highlight the disc’s broad adaptability and robust adhesion in real-world scenarios, both in air and under water.

### Cross-medium adhesion demonstration

**Fig. 6.**
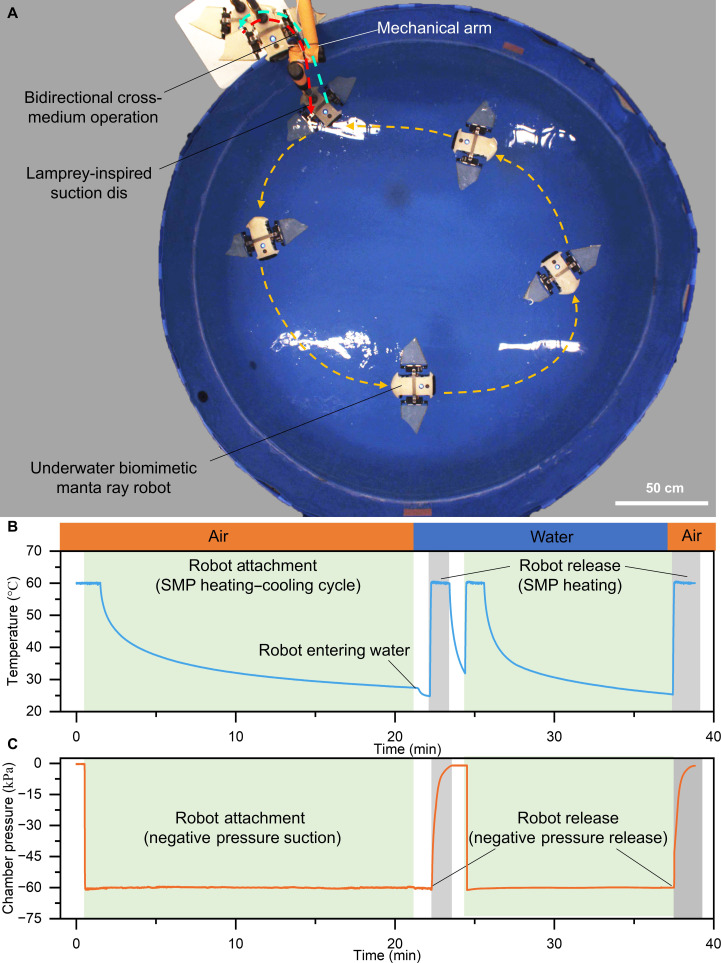
Cross-medium robotic manipulation using the lamprey-inspired suction disc. (A) Demonstration of bidirectional cross-medium operation. A mechanical arm equipped with the lamprey-inspired suction disc attaches to an underwater biomimetic manta ray robot in air, immerses it into water, and then retrieves it back to air, showcasing robust reversible adhesion across media (Movie S4). (B) Time-resolved temperature profile of the SMP panel during the operation. The robot attaches through an SMP heating–cooling cycle, enters the water, and is released by reheating the SMP. (C) Corresponding chamber pressure (relative to ambient) showing stable vacuum during attachment and controllable release through negative pressure venting. Blue shading marks the attachment phase and gray shading marks the release phase.

To further validate the system-level functionality of the lamprey-inspired suction disc, we integrated it with a mechanical arm to demonstrate bidirectional cross-medium adhesion and release. The robotic setup grasped an underwater biomimetic manta ray robot in air, immersed it into water, and subsequently retrieved it back into air (Fig. 6A and Movie S4). This experiment highlights the disc’s ability to maintain reliable adhesion during transitions across air and water interfaces and to achieve controllable detachment.

Time-resolved temperature and chamber gauge pressure (relative to ambient) were recorded simultaneously to capture the coupled behavior of the SMP and vacuum systems (Fig. 6B and C). During attachment, the SMP was first heated above its glass transition temperature (60 °C) to achieve conformal contact, followed by cooling to lock the interfacial geometry. In parallel, the chamber pressure was actively regulated to approximately −60 kPa, providing the SMP with the required preload and initial adhesion force. Notably, although cooling of the SMP could in principle deepen the vacuum, the pressure trace showed no appreciable further drop, indicating that any thermal effect on chamber pressure was negligible. During release, reheating the SMP and venting the chamber back toward ambient enabled rapid detachment. The blue-shaded regions in Fig. 6B and C denote attachment, whereas the gray-shaded regions indicate release. Together, these synchronized temperature and pressure profiles confirm the cooperative action of SMP phase transition and negative pressure suction for stable, reversible cross-medium adhesion.

## Discussion

In this work, we present a lamprey-inspired suction disc that couples negative pressure suction with a thermally switchable shape-memory interlocking layer to enable strong, reversible attachment across media. The disc consists of a soft silicone lip that seals the interface, an SMP pillar array that provides switchable interlocking, and an embedded flexible heater that controls the SMP state. During attachment, vacuum rapidly establishes suction and simultaneously supplies a uniform preload that presses the softened SMP into surface asperities to form an imprint. Cooling then restiffens the SMP and locks the imprinted geometry to strengthen attachment, whereas reheating restores compliance for on-demand release. Together, suction-driven sealing and SMP-enabled locking create a reusable adhesion cycle inspired by the lamprey oral discs with tooth-like microstructures.

The developed suction disc demonstrates rapid thermal actuation and strong adhesion in both air and underwater environments. Dynamic mechanical analysis revealed a distinct SMP glass transition around 33 °C. The integrated heating circuit raised the SMP temperature to 60 °C within 2.8 s in air and 5.4 s underwater. On smooth substrates at a vacuum pressure difference of −60 kPa, the disc achieved maximum pull-off forces of 562.6 ± 8.0 N in air and 590.7 ± 3.5 N underwater, corresponding to adhesion stresses up to 78.3 kPa. Even on highly rough surfaces (*R*_a_ = 707 μm), the disc retained strong adhesion, outperforming vacuum-only systems. At moderate roughness (*R*_a_ = 25 μm), incorporating the SMP layer increased adhesion forces by 377% in air and 270% underwater compared to a control without the SMP. The frictional performance showed similar improvement, with peak shear stress reaching 58.6 ± 0.4 kPa underwater. Under sustained loading (5 N on a *R*_a_ = 25 μm), the disc remained attached for up to 26.8 ± 0.5 h, representing a 540% increase in underwater retention time compared with the control.

Controlled tests confirmed that the vacuum-SMP hybrid mechanism acts synergistically to enhance adhesion performance. Beyond generating suction, negative pressure benefits the SMP layer in 2 ways. First, it provides a spatially uniform compressive preload that drives the softened SMP into surface asperities, improving conformity and imprint formation without external pressing. Second, this distributed preload stabilizes the interface and reduces local stress concentrations, increasing the effective adhesion stress that can be sustained after SMP locking. In turn, the SMP layer markedly reinforces both adhesion and friction compared with a vacuum-only disc. It prolongs attachment, allowing the disc to remain adhered long after the negative pressure has dissipated. The complementary interaction between the vacuum preload and the thermally switchable SMP interlocking yields strong, durable, and reversible adhesion across a wide range of surface types.

**Fig. 7.**
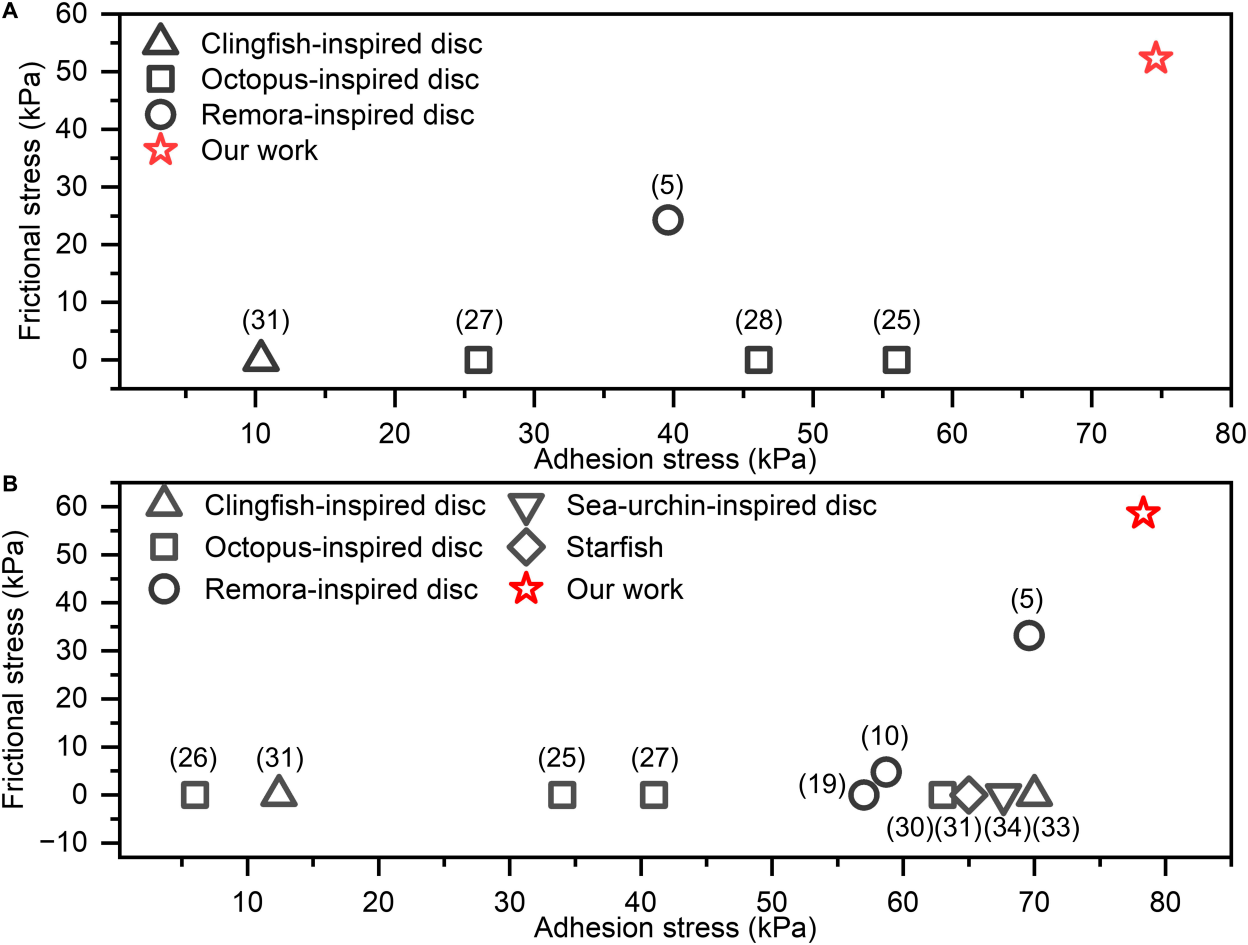
Comparison of adhesion and frictional stresses of the lamprey-inspired suction disc with previously reported bioinspired suction systems. (A) In air and (B) underwater. Each symbol represents reported maximum stresses of different bioinspired suction discs on smooth surfaces; zero values indicate unmeasured friction data. The red star marks the performance of the present lamprey-inspired disc. Numbers in parentheses indicate the reference numbers.

To benchmark performance, we compared our lamprey-inspired suction disc with representative bioinspired systems, including remora- [5,10,19], octopus- [25–29], starfish- [30], clingfish- [31,32], and sea-urchin-inspired discs [33], both in air and underwater (Fig. 7 and Table S1). Figure 7 highlights the most directly comparable metrics, adhesion, and friction stresses, whereas Table S1 compiles additional context, including operating medium, adhesion mechanism, adhesion duration, surface adaptability (roughness and curvature), response time, and qualitative energy requirements. In air, prior designs report adhesion stresses up to 56 kPa (octopus disc) and friction stresses up to 24.3 kPa (remora disc), both below our values. Underwater, reported adhesion stresses reach 70 kPa (clingfish disc), and friction stresses reach 33.2 kPa (remora disc), again lower than those achieved by our hybrid disc. These comparisons highlight the advantages of integrating shape-memory-assisted interlocking with negative pressure suction, providing a substantial improvement over traditional pressure-based adhesion strategies. Beyond peak stresses, Table S1 further shows that our disc offers the strongest overall surface adaptability and long-duration attachment under load across challenging substrates within the set of systems compared. This performance comes with an operational trade-off. Because the SMP layer must be heated and then cooled to lock the imprinted interface, the response time is longer, and the energy demand is higher than purely pressure-driven suction systems. Even so, the energy requirement is lower than SMP-only interlocking approaches, which typically rely on heating together with sustained external preload during cooling to maintain intimate contact for locking [34].

This work establishes a multifunctional, environment-adaptive adhesion module that supports reliable attachment and controllable release in air and underwater settings, including during air–water transitions. The fabrication workflow is scalable because it relies on molding and layered assembly rather than size-specific microfabrication, and the design can be resized by tuning the disc diameter, lip geometry, and cavity volume. With miniaturization, absolute pull-off force will decrease with area, but stress-level performance can be maintained if sealing and SMP locking remain effective. The same architecture can be integrated as a compact end-effector or distributed attachment unit across robotic platforms, with pumping and heating packaged on-board or supplied remotely. In arrays, localized attachment and release can be achieved by independently addressing vacuum and heating for each disc (or small groups), which also helps limit thermal and mechanical cross-talk. A remaining limitation is the cooling rate of the SMP, which currently constrains reattachment speed during dynamic operation. Future work will therefore prioritize thermal engineering, for example, by integrating high-conductivity heat-spreading layers and compact heat sinks or fluid-assisted cooling. Together, these advances should enable rapid, repeated attachment cycles and tighter integration into autonomous robotic manipulators, climbing robots, and amphibious systems.

## Data Availability

All data are available in the main text or the Supplementary Materials.
