# Supplementary material for: Lamprey-Inspired Amphibious Suction Disc with Hybrid Adhesion Mechanism
Source: Cyborg Bionic Syst. 2026 Feb 24;7:0527. doi: 10.34133/cbsystems.0527 (PMC12929816; doi:10.34133/cbsystems.0527)
Supplement: Supplementary 1 — Text S1 Figs. S1 to S6 Table S1 Movies S1 to S4 [file cbsystems.0527.f1.zip › Supplemental Materials.docx]

**Supplemental Materials**

Title

Lamprey-inspired amphibious suction disc with hybrid adhesion mechanism

**Authors**

Lei Li^1,2^†, Wenzhuo Gao^2^†, Boyang Qin^2^, Yiyuan Zhang^3^, Changhong Linghu^4^, Bo Wang^2^, Yitian Ma^5^, Shihan Kong^2^, Li Wen^6^, Junzhi Yu^2^*

**Affiliations**

^1^Institute of Ocean Research, Peking University, Beijing, China.

^2^College of Engineering, Peking University, Beijing, China.

^3^College of Design and Engineering, National University of Singapore, Singapore, Singapore.

^4^Department of Mechanical Engineering, City University of Hong Kong, Hong Kong SAR, China.

^5^School of Mechanical and Electrical Engineering, Beijing Institute of Technology, Beijing, China.

^6^School of Mechanical Engineering and Automation, Beihang University, Beijing, China.

*Address correspondence to: yujunzhi@pku.edu.cn

†These authors contributed equally to this work.

**This file includes:**

Supplementary Text 1

Figs. S1 and S6

Table S1

**Supplementary Text 1**

**Logic Description of the Control Algorithm for the Suction System**

The control logic for the suction system is implemented as a multi-modal state machine designed to facilitate precise coordination between thermal regulation and pneumatic actuation. To ensure high-fidelity feedback, the system architecture prioritizes signal integrity through an intensive data acquisition routine. Raw voltage data from the sensor array undergo a 1024 oversampling and array averaging process to mitigate high-frequency electronic noise and enhance the effective resolution of the thermal measurements. These processed signals are subsequently converted into real-time temperature *T_cur_* and pressure *P_cur_* values using pre-calibrated lookup tables, while simultaneous monitoring of the heater’s electrical status provides a telemetry stream for continuous system diagnostics and experimental recording at a loop frequency of 20 Hz (sampling interval *Δt* = 50ms).

Safety and hardware longevity are maintained through a low-latency interlock mechanism that resides at the highest priority of the execution cycle. By continuously evaluating the bus voltage *V_bus_* and current *I_bus_* against predefined safety thresholds, the algorithm can instantaneously truncate the pulse-width modulation (PWM) output *u* to the FPC heaters in the event of an electrical anomaly. This protective layer is decoupled from the primary control laws, ensuring that thermal runaway or over-current conditions are averted regardless of the current operational state or command input.

The functional behavior of the system is governed by three distinct operational modes that manage the phase transitions of the shape memory polymer and the vacuum state of the suction interface. In the heating-adhesion mode, a PID control law characterized by gains {*K_p_*, *K_i_*, *K_d_*} drives the system toward the target temperature *T_set_*, while the vacuum valve *S_vac_* is dynamically regulated to maintain the required negative pressure *P_set_*. Conversely, the cooling- adhesion mode disables active heating to allow for passive cool down while maintaining the vacuum seal. Finally, the desorption mode coordinates thermal maintenance with the activation of a venting valve *S_vent_*, effectively neutralizing the internal vacuum to facilitate the rapid release of the suction unit. This integrated approach ensures that the mechanical state of the suction system is strictly synchronized with the glass transition of the SMP materials.

| **Algorithm 1:** Control Logic for the Thermally Actuated Suction System |
| --- |
| **Require:** Target Temp *Tset* , Target Vacuum *Pset*  **Require:** Safety Thresholds *Vmax* , *Imax*, Sampling interval *Δt*  **Ensure:** Heater PWM *u*, Vacuum Valve *Svac* , Vent Valve *Svent*  1: **Initialize:** *u* ← 0, *eprev* ← 0, Integral *I* ← 0  2: **while** System is Active **do**  3: // *1 . Data Acquisition & Communication*  4: Receive *Cmd* via Serial/Wireless Interface  5: Sample Temperature Sensor *Vt* [4][1024] ▷ Oversampling  6: Calculate Average: *Vavg* ←$\frac{1}{4\times1024}\sum_{i=0}^{3} \sum_{j=0}^{1023} V_{t}\left[i\right]\left[j\right]$  7: *Tcur* ← LookupTableT (*Vavg* )  8: *Pcur* ← LookupTableP (ReadPressureSensorVoltage())  9: Read Heater Status *Vbus* , *Ibus*  10: **Transmit** State {*Tcur* , *Pcur* , *Vbus* , *Ibus* } via Serial Port ▷ Telemetry  11: // *2. Safety Interlock*  12: **if** *Vbus* > *Vmax* **or** *Ibus* > *Imax* **then**  13: *u* ← 0  14: *Svac* ← CLOSED  15: *Svent* ← OPEN  16: **Update** PWM(*u*), Valves(*Svac* , *Svent* )  17: **continue** loop  18: **end if**  19: // *3. State Machine Execution*  20: **if** *Cmd* = **MODE HEAT ATTACH then**  21: *e* ← *Tset* - *Tcur*  22: *u* ← *Kpe* + *KiI* + *Kd* (*e* - *eprev* ) ▷ Active Heating  23: *Svent* ← CLOSED  24: **if** *Pcur* < *Pset* **then** *Svac* ← OPEN  25: **else** *Svac* ← CLOSED  26: **end if**  27: **else if** *Cmd* = **MODE COOL ATTACH then**  28: *u* ← 0 ▷ Passive Cooling  29: *Svent* ← CLOSED  30: **if** *Pcur* < *Pset* **then** *Svac* ← OPEN ▷ Maintain Vacuum  31: **else** *Svac* ← CLOSED  32: **end if**  33: **else if** *Cmd* = **MODE DETACH then**  34: *e* ← *Tset* - *Tcur*  35: *u* ← *Kpe* + *KiI* +*Kd* (*e* - *eprev* ) ▷ Heating for Detachment  36: *Svac* ← CLOSED  37: *Svent* ← OPEN ▷ Release Pressure  38: **end if**  39: // *4 . Actuation Update*  40: **Update** PWM(*u*), Valves(*Svac* , *Svent* )  41: *eprev* ← *e*  42:  *I* ← *I + eΔt*  43: Wait 50 ms  44: **end while** |


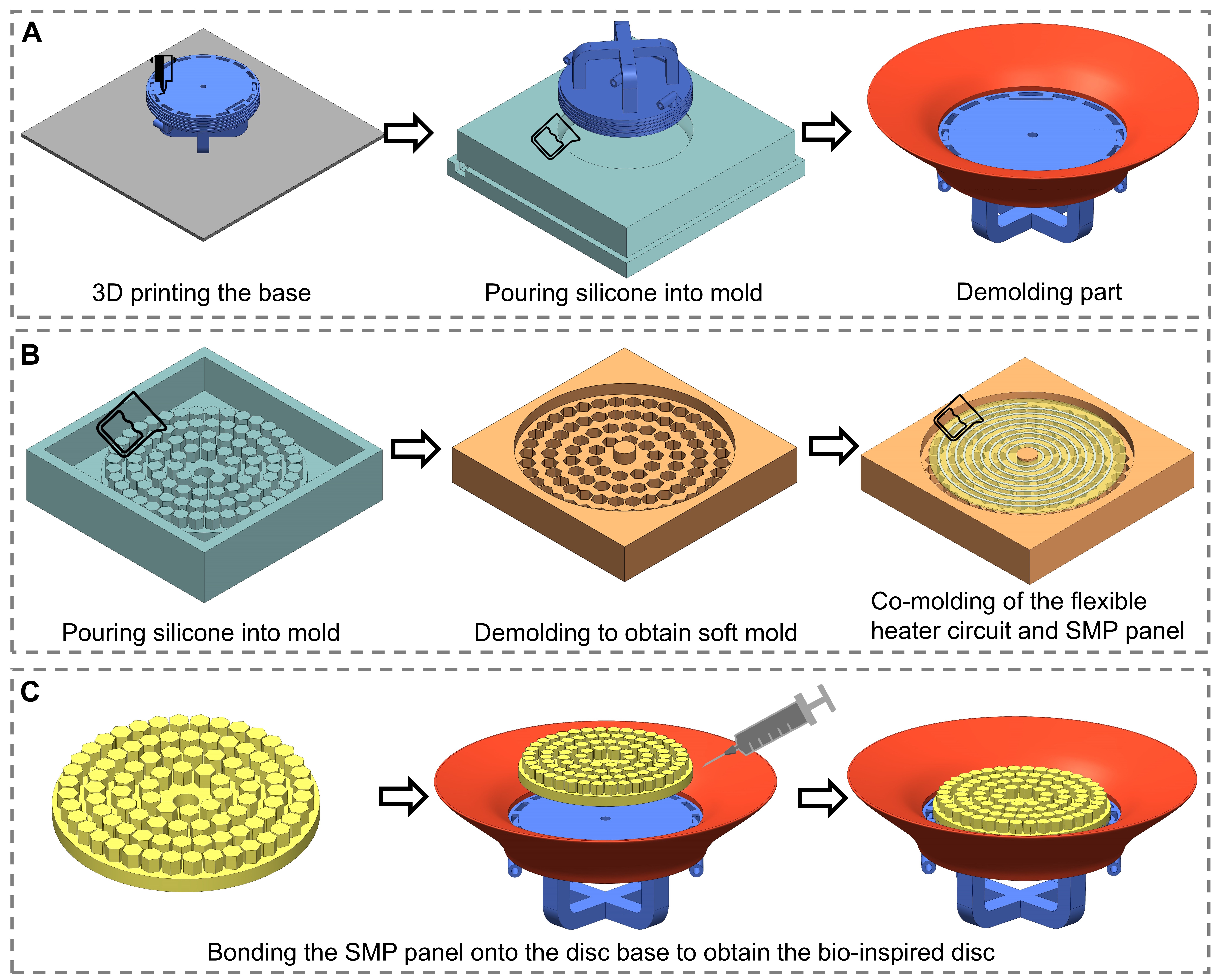


**Fig. S1.** **Fabrication of the bio-inspired suction disc**. (A) Cast a soft silicone lip (Ecoflex 30) onto a 3D-printed disc base. (B) Use the 3D-printed mold to form an inverted soft mold and co-mold the flexible heater circuit with the SMP panel. (C) Demold the cured SMP panel, bond it to the disc base, and complete the bio-inspired suction disc.





**Fig. S2.** **Architecture and hardware implementation of the integrated heating-vacuum control system.** (A) Schematic of the electrical control system. (B) Photographs of the control electronics and the FPC heating element, highlighting the MCU board, power/heating interfaces, valve/pressure interfaces, wireless module and antenna, and the heater coil with a backside temperature sensor.


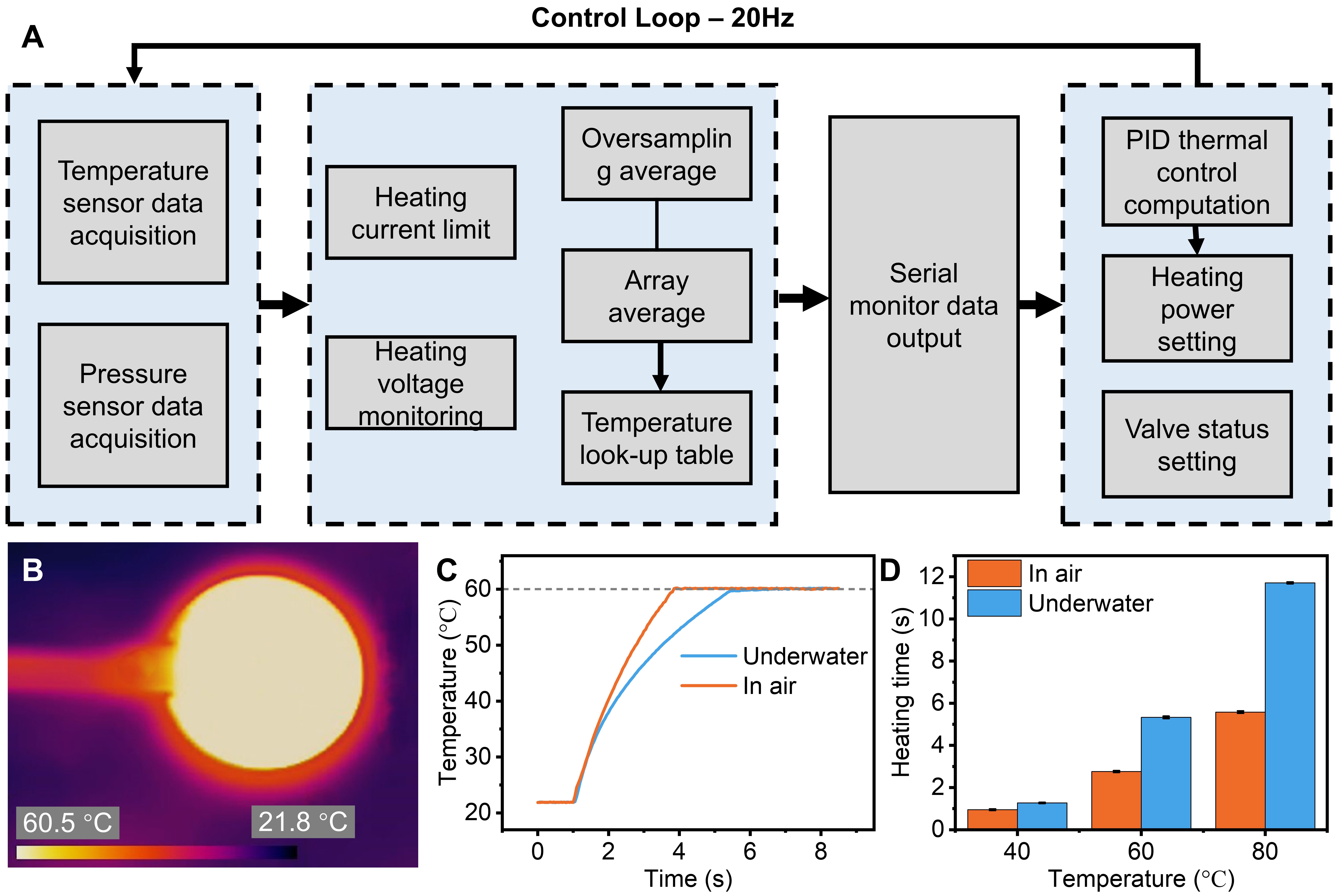


**Fig. S3.** **Closed-loop control and thermal control of the suction disc.** (A) Block diagram of the 20 Hz closed-loop controller for temperature and pressure regulation. (B) Thermal image of the SMP panel heated to 60 °C. (C) Temperature versus time in air and underwater at a 60 °C setpoint. (D) Comparison of heating times for 40 °C, 60 °C, and 80 °C setpoints.


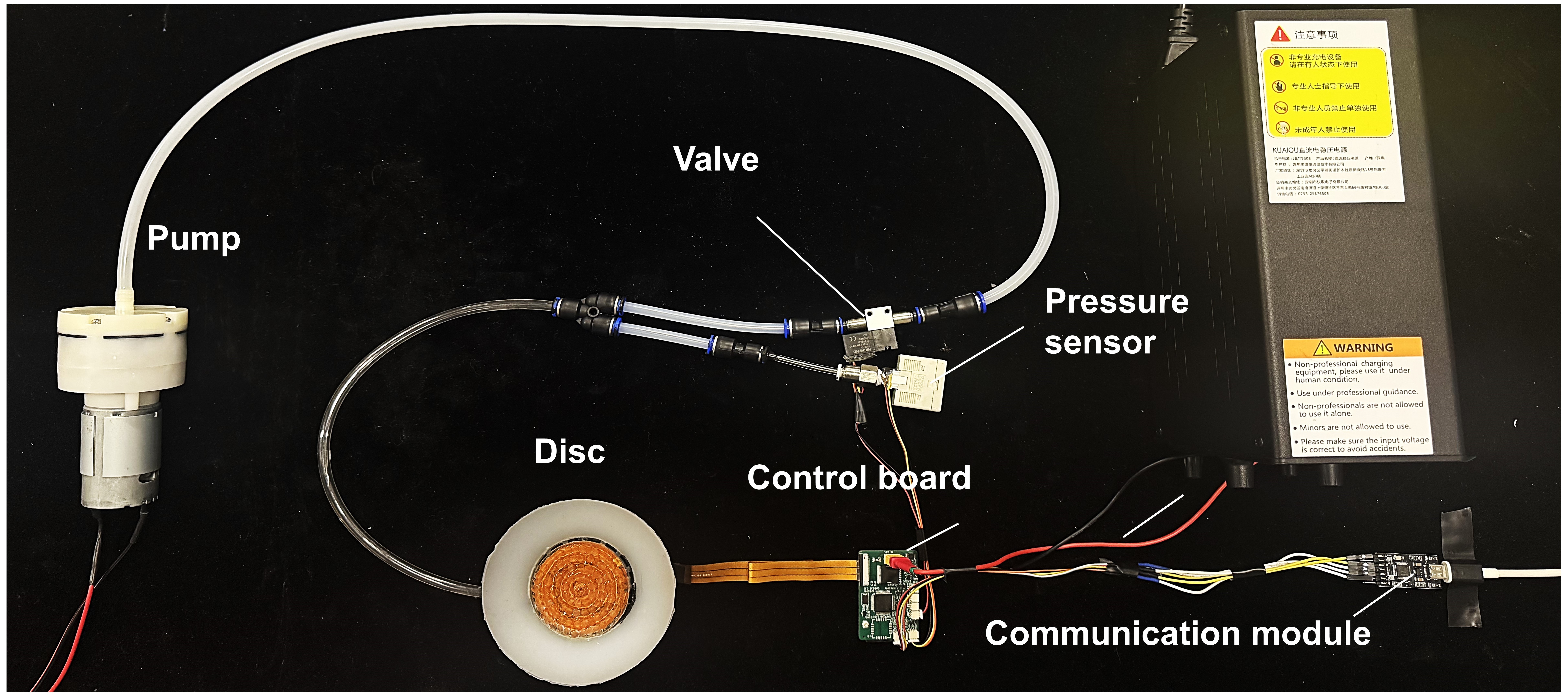


**Fig. S4. Experimental setup of the integrated suction system, consisting of a biomimetic suction disc, motor-driven negative-pressure module, controller, and data acquisition unit.**


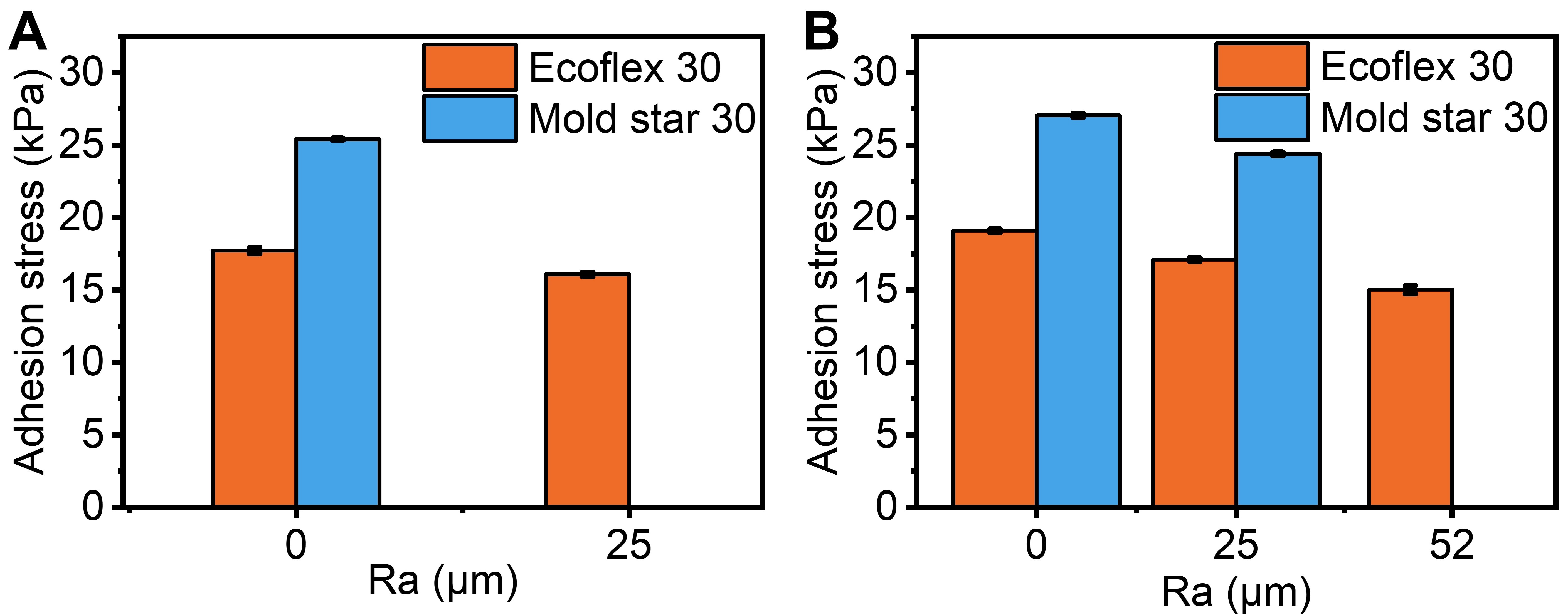


**Fig. S5.** **Effect of silicone stiffness on suction-cup adhesion across surface roughness (no SMP).** (A) Adhesion stress measured in air. (B) Adhesion stress measured underwater. Suction cups cast from two silicones with different stiffness (Ecoflex 30, softer; Mold Star 30, stiffer) were tested on substrates with different roughness (*N* = 5).


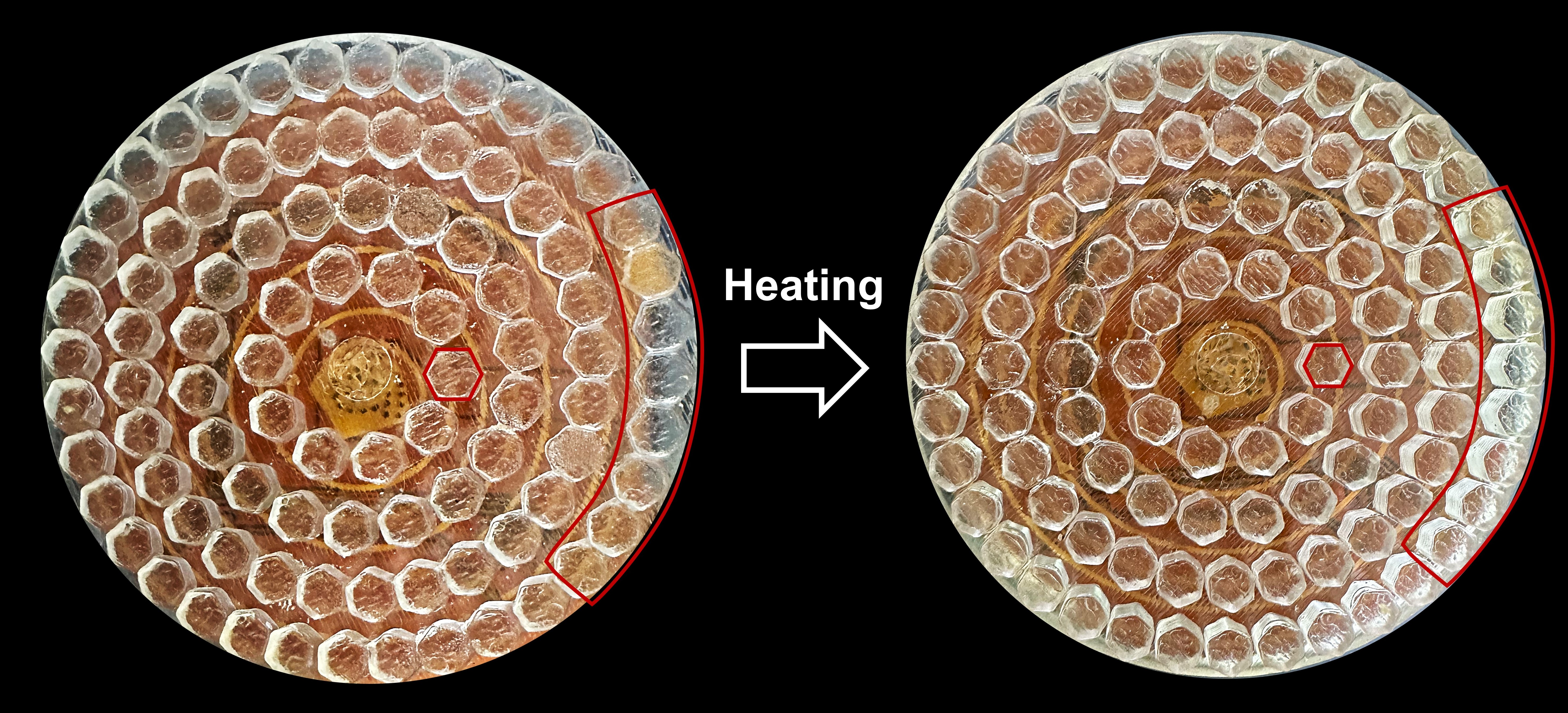


**Fig. S6. Thermal recovery of SMP array panel after long-term adhesion.** Prolonged adhesion causes the SMP pillar array to deform, with pillars shortened and their cross-sections enlarged (left; highlighted region). Upon heating, the SMP recovers its original geometry and the pillars return toward their initial shape (right).

**Table S1. Performance comparison of bio-inspired suction discs and SMP-enabled adhesion.**

| System (type) | Medium | Adhesion mechanism | Adhesion stress (air) | Friction stress (air) | Adhesion stress (underwater) | Friction stress (underwater) | Adhesion duration | Surface adaptability (roughness/radius of curvature) | Response time | Energy consumption (qualitative) | Reference |
| --- | --- | --- | --- | --- | --- | --- | --- | --- | --- | --- | --- |
| **lamprey -inspired disc** | **Air / water** | **Negative-pressure suction and SMP interlocking** | **74.6 kPa** | **52.3 kPa** | **78.3 kPa** | **58.6 kPa** | **1608 min (25µm substrate, load 500 g)** | **> 707 µm/*R*_min_ < 1 mm** | **About 5 min** | **L3: pumping and heating** | **Our work** |
| Clingfish-inspired disc | Water | Negative-pressure suction | N/A | N/A | 70 kPa | N/A | 3 weeks (rough substrate, no load) | 269 μm/N/A | < 5 s | L1: press-to-seal | 31 |
| Clingfish-inspired disc | Air / water | Negative-pressure suction | 10.4 kPa | N/A | 12.4 kPa | N/A | 383 min (3µm substrate, load 182 g) | 269 μm/*R*_min_ = 12.5 mm | < 5 s | L1: press-to-seal | 32 |
| Octopus-inspired disc | Air / water | Negative-pressure suction and chemical adhesion | 56 kPa | N/A | 34 kPa | N/A | N/A | N/A | < 5 s | L1: press-to-seal | 25 |
| Octopus-inspired disc | Water | Negative-pressure suction | N/A | N/A | 6 kPa | N/A | N/A | N/A | < 10 s | L2: pumping | 26 |
| Octopus-inspired disc | Air / water / oil | Negative-pressure suction | 26 kPa | N/A | 41 kPa | N/A | N/A | N/A | < 5 s | L1: press-to-seal | 27 |
| Octopus-inspired disc | Air | Negative-pressure suction | 46.1 kPa | N/A | N/A | N/A | N/A | 269 μm/N/A | < 10 s | L2: pumping | 28 |
| Octopus-inspired disc | Air / water | Negative-pressure suction | 60 kPa | N/A | 60 kPa | N/A | N/A | N/A | <0.1s | L2: pumping | 29 |
| Remora-inspired disc | Air / water | Negative-pressure suction | 39.6 kPa | 24.3 kPa | 69.6 kPa | 33.2 kPa | 2944.4 min (50 µm substrate, load 1000 g) | 764 μm/*R*_min_ = 20 mm | < 10 s | L2: pumping | 5 |
| Remora-inspired disc | Water | Negative-pressure suction and microstructured friction | N/A | N/A | 58.7 kPa | 4.77 kPa | N/A | 200 μm/Not supported | < 10 s | L2: pumping | 10 |
| Remora-inspired disc | Water | Negative-pressure suction | N/A | N/A | 57 kPa | N/A | 26 min (50 µm substrate, load 5000 g) | 200 μm/Not supported | < 10 s | L1: press-to-seal | 19 |
| Sea-urchin-inspired disc | Water | Negative-pressure suction and mucus | N/A | N/A | 67.65 kPa | N/A | N/A | N/A/Not supported | < 10 s | L2: pumping | 33 |
| Starfish- inspired disc | Water | Negative-pressure suction | N/A | N/A | 65 kPa | N/A | >1 h | N/A | < 5 s | L1: press-to-seal | 30 |
| SMP | Water / oil | SMP interlocking | N/A | N/A | 1.82 MPa | N/A | N/A | N/A | About 5 min | L4: heating and sustained preload | 34 |

Table notes: Energy consumption is reported qualitatively by actuation actions. L1 (passive suction): one-time mechanical pressing to form a seal, without pumping or heating. L2 (active suction): pumping/pressure regulation to generate negative pressure. L3 (hybrid, this work): active suction provides vacuum-induced preload and sealing, while heating is applied only transiently to switch the SMP state; after cooling, interfacial locking reduces reliance on sustained vacuum. L4 (SMP-only locking): requires heating and externally applied sustained preload throughout the locking step, imposing the highest overall actuation burden. N/A indicates not reported
